# Tolerance of sonidegib after intolerance of vismodegib—Experience in two patients with nevoid basal cell carcinoma syndrome (Gorlin syndrome)

**DOI:** 10.1002/ski2.260

**Published:** 2023-06-14

**Authors:** Federico Venturi, Luciana Trane, Flavia Silvestri, Biancamaria Zuccaro, Elisabetta Magnaterra, Vincenzo De Giorgi

**Affiliations:** ^1^ Section of Dermatology Department of Health Sciences University of Florence Florence Italy; ^2^ IRCCS Azienda Ospedaliero-Universitaria di Bologna Bologna Italy; ^3^ Dermatology Unit, Department of Medical and Surgical Sciences (DIMEC), Alma Mater Studiorum University of Bologna Bologna Italy; ^4^ Cancer Research “Attilia Pofferi” Foundation Pistoia Italy

## Abstract

Nevoid basal cell carcinoma syndrome (NBCCS, Gorlin syndrome) is a rare genetic condition characterized by the early development of numerous cutaneous basal cell carcinomas (BCCs). Although most BCCs are surgically treated with total resection, some of the lesions may proceed to a locally advanced or metastatic stage. Systemic treatment with a hedgehog inhibitors (HHIs) such as Vismodegib or Sonidegib is indicated in this population. We report cases of two patients with confirmed diagnoses of NBCCS. Both patients had undergone multiple surgical excisions and had been treated with oral Vismodegib 150 mg/day for a locally advanced tumour. They both discontinued the therapy due to its specific adverse effects (AEs) and are now being treated with oral Sonidegib, which has had better tolerability and a complete response. The aims of this report was to demonstrate the efficacy of HHI treatment with Sonidegib in patients with NCBBS who had previously treated with Vismodegib but discontinued it because of its specific AEs. Our experience in two patients shows that Sonidegib can be considered in Gorlin patients intolerant but responding to Vismodegib.

## Graphical abstract

We report cases of two patients with confirmed diagnoses of Gorlin Syndrome. Both patients had been treated with oral Vismodegib 150 mg/day for a locally advanced tumour. They both discontinued the therapy due to its specific AEs and are now being treated with oral Sonidegib, which has had better tolerability and a complete response.

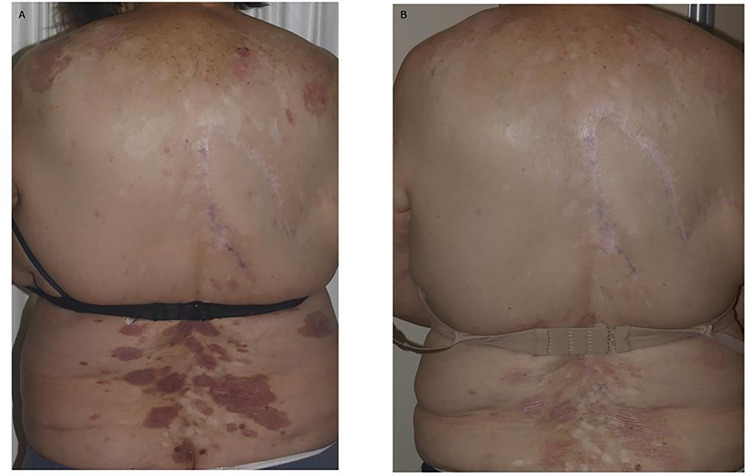

## 1 INTRODUCTION

Also known as Gorlin syndrome (GS), Nevoid basal cell carcinoma syndrome (NBCCS) is a rare genetic condition characterized by the early development of numerous cutaneous basal cell carcinomas (BCCs).^1^ The most prevalent cause of NBCCS is loss‐of‐function germline mutations in the hedgehog‐related Patched 1 (PTCH1) tumour suppressor gene.^1^ The prevalence of NBCCS ranges from 1/57 000 to 1/256 000, with an equal sex distribution and roughly 20%–30% de novo mutation.^2^, ^3^, ^4^ The frequency of BCCs is rising, particularly in children, and it often requires recurrent surgical excisions, sometimes resulting in deformity and psychological trauma.^5^, ^6^ However, due to the vast number of tumours, treating BCCs in patients with NBCCS can be particularly challenging.

Although most BCCs are surgically treated with total resection, some of the lesions may proceed to a locally advanced or metastatic stage. Systemic treatment with a hedgehog inhibitors (HHIs) such as Vismodegib or Sonidegib is indicated in this population.^7^, ^8^, ^9^ The chemical structures of Vismodegib and Sonidegib are different as they bind different residues of the same functional pocket, but they both suppress hedgehog signalling through selective inhibition of smoothened. Sonidegib, in particular, features a substantially greater concentration in the skin and other tissues due to its high lipophilicity and a lower plasmatic concentration.^10^, ^11^ Furthermore, both treatments have been studied in patients with NBCCS, and the objective response rate is around 60% of patients, although there are links with poor tolerability due to their specific side effects.^12^ The conventional adverse effects (AEs) associated with therapy include muscular cramps, alopecia, dysgeusia, nausea, fatigue which may lead to treatment withdrawal, especially in a long‐term treatment of patients who receive diagnosis at a young age. We report cases of two patients with confirmed diagnoses of NBCCS. Both patients had undergone multiple surgical excisions and had been treated with oral Vismodegib 150 mg/day for a locally advanced tumour. They both discontinued the therapy due to its specific AEs and are now being treated with oral Sonidegib, which has good tolerability and good tumour response.

## 2 CASE REPORT

### 2.1 Case 1

A 54‐year‐old male (height 1.75 m; weight 84 kg) with NBCCS was evaluated for a tumour located on the left nasal fold, which caused ongoing bleeding and impaired his quality of life. In 2003, at the age of 35, NBCCS was diagnosed based on the presence of three major criteria (multiple BCCs, >3 palmar pits, and first degree relative with NBCCS) and confirmed by the detection of Patched mutation. According to the patient's medical history, he had previously undergone multiple surgical excisions and had been effectively treated with Vismodegib 150 mg/day for the same lesion, as well as multiple pigmented BCCs located on the trunk. However, Vismodegib was discontinued after 13 months due to AEs such as G2 muscular cramp with CPK rise, G2 alopecia, and moderate dysgeusia.

**FIGURE 1:**
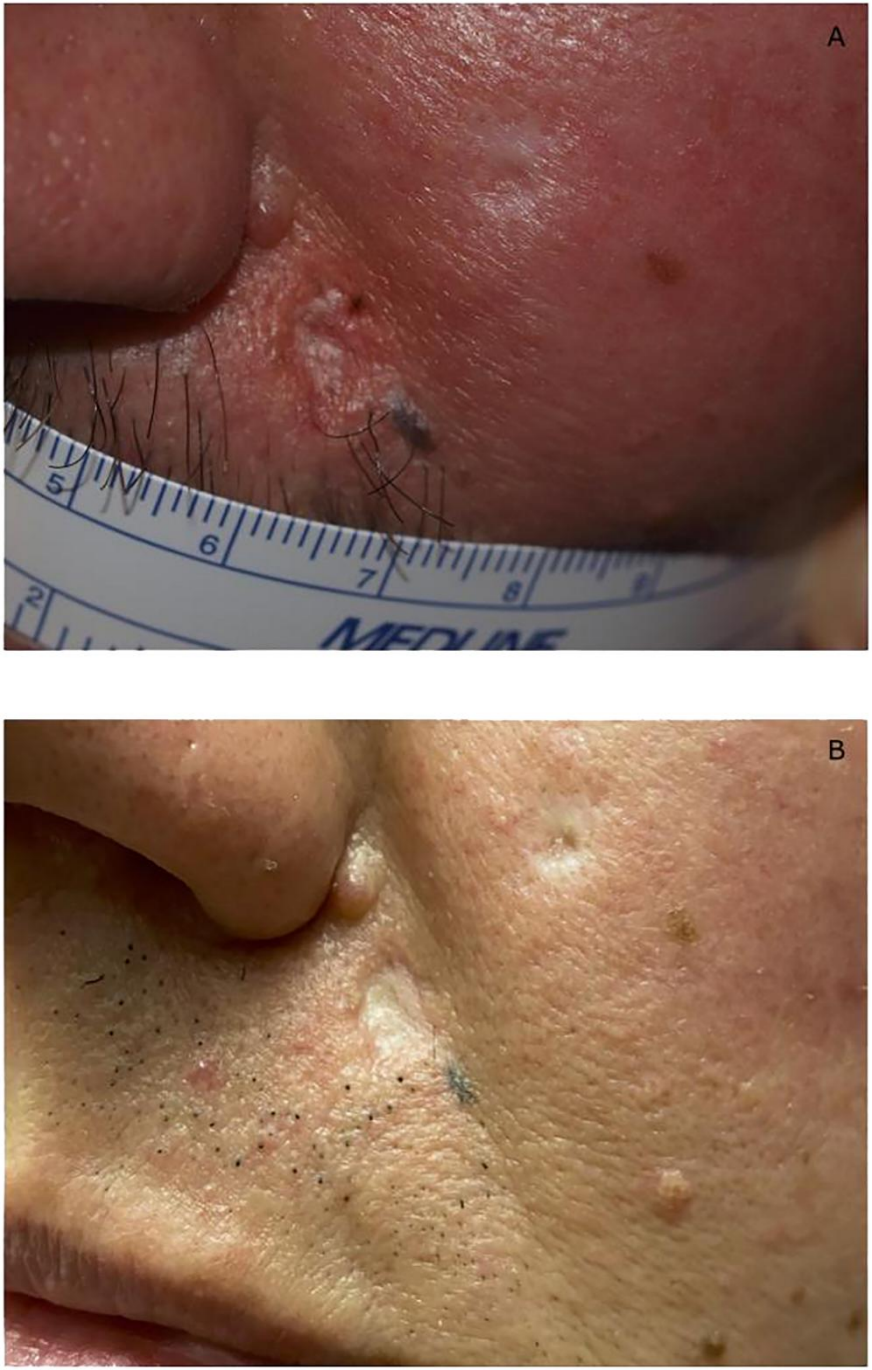
Case 1. Clinical presentation of cutaneous BCC located on the left nasal fold in a 54‐year‐old patient with NBCCS: (a) pre‐treatment, (b) efficacy after 15 months of treatment with Sonidegib 200 mg/die. BCC, basal cell carcinoma; NBCCS, nevoid basal cell carcinoma syndrome.

The patient was lost to follow‐up, but 48 months after the interruption of Vismodegib, he was seen again due to relapse of the treated BCCs, specifically an ulcerated lesion on the left nasal fold and upper lip (Figure 1a), as well as multiple superficial BCCs on the trunk.

According to the Italian Medicines Agency (AIFA), we started oral treatment with Sonidegib 200 mg/day in May 2021. After 3 months, we detected partial re‐epithelization in all the target lesions, and no drug‐related AEs were reported. After 15 months, there was further response, with complete response of the BCC on the face (Figure 1b) and partial response of the BCCs on the trunk. At present, Sonidegib medication has been maintained with clinical benefit and no drug‐related AEs except for G1 muscular cramp with no CPK increase and no dosage adjustment. During the follow‐up visits, laboratory test revealed no abnormalities.

### 2.2 Case 2

**FIGURE 2:**
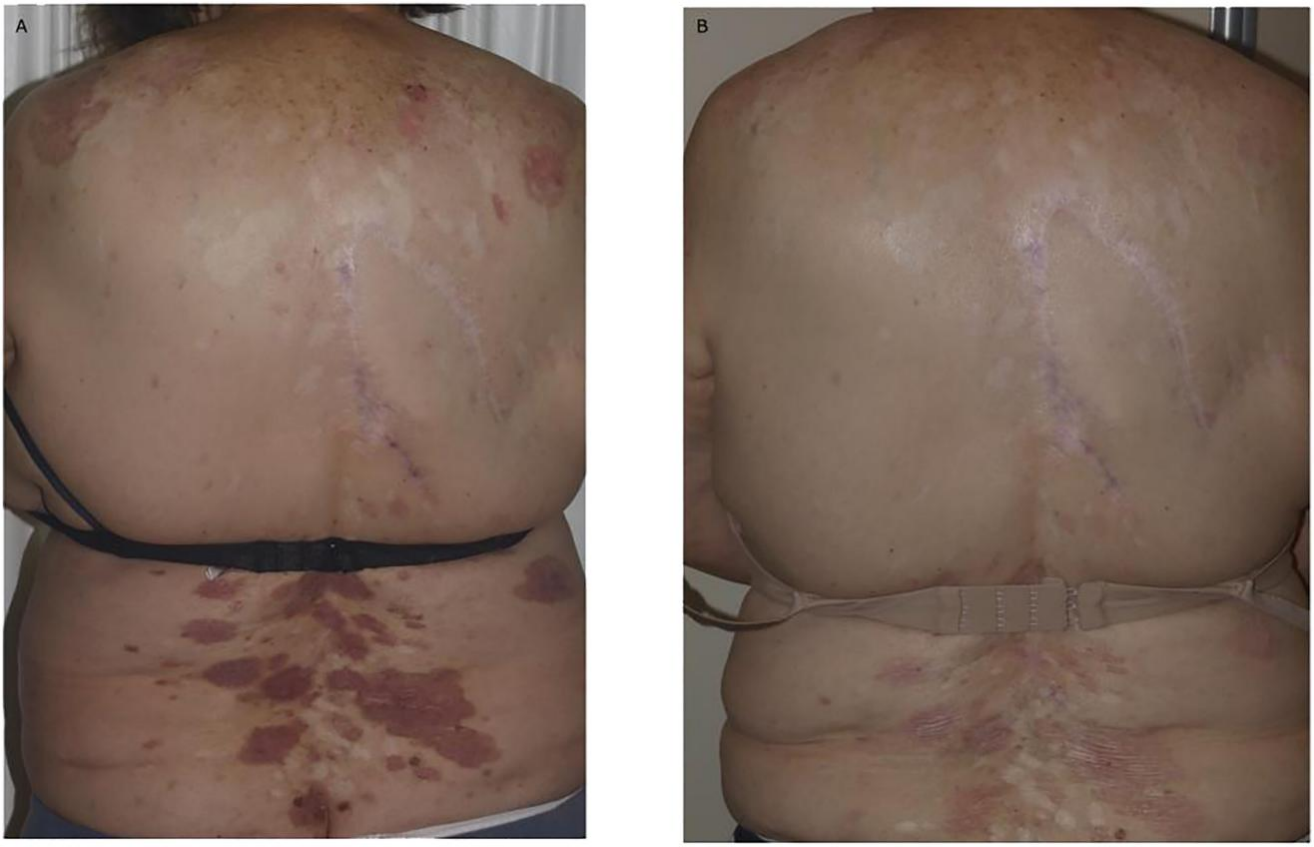
Case 2. Clinical presentation of diffuse spreading BCCs located on the back in a 74‐year‐old woman with NBCCS: (a) pre‐treatment; (b) efficacy after 6 months of treatment with Sonidegib 200 mg/die. BCC, basal cell carcinoma; NBCCS, nevoid basal cell carcinoma syndrome.

A 74‐year‐old woman (height 1.64 m; weight 82 kg) with a long‐term history of NBCCS was referred to our clinic for diffuse spreading BCCs located on the back. The first diagnosis of NBCCS was made in the late 1980s. She had no comorbidities and was not using any long‐term medications. The patient had been treated for several BCCs with surgery, cryotherapy, and topical 5% imiquimod from the age of 12 years. Most lesions were located on the trunk with some on the head and neck area. Furthermore, the patient was given Vismodegib 150 mg/day for 36 months with a complete response by the targeted lesions. However, treatment with oral Vismodegib was halted due to AEs of G2 alopecia, G2 muscular spasm, G1 dysgeusia, and nausea. In December 2021, after 18 months of drug discontinuation, the patient presented to our clinic for diffuse spreading BCCs located on the back which had relapsed after HHI discontinuation (Figure 2a). After case discussion in a multidisciplinary board, 200 mg/day Sonidegib was selected in consideration of the lower risk of drug resistance and the possibility of modifying the dose in case of AEs. The number and size of the lesions decreased after 4 months of Sonidegib. The patient only had grade 1 alopecia as an AE. Laboratory testing during follow‐up revealed no abnormalities. By June 2022, after 6 months of therapy, all lesions had a full clinical response (Figure 2b). Sonidegib therapy is being continued with clinical benefit and no drug‐related AEs due to high tolerability, and the patient has had a full clinical response. The patient is satisfied with Sonidegib medication and does not require every‐other‐day doses.

## 3 DISCUSSION

The antagonistic effect on Hedgehog signalling is a major treatment opportunity for patients with locally advanced, metastatic BCCs as well as patients with NBCCS who have a high risk of developing numerous BCCs throughout their lives.^7^, ^8^, ^9^, ^13^ Vismodegib and Sonidegibare both indicated for this peculiar subset of patients. In clinical trials, these drug have shown comparable clinical effectiveness, safety, and tolerance characteristics.^12^ Pharmacokinetic profiles reveal most of the biological differences between the two HHIs. The half‐life of Sonidegib is longer than that of Vismodegib (29.6 vs. 4–12 days), and both drugs bind to plasma proteins very strongly, with Vismodegib being concentration‐dependent and Sonidegib being concentration‐independent.^14^, ^15^ To date, there has been no direct comparison of Vismodegib and Sonidegib in a randomized controlled clinical study. A panel of European experts recently determined that the ERIVANCE and BOLT trials were adequate for indirect comparisons of Sonidegib and Vismodegib. These studies found an objective response rate (ORR) of 47.6% for Vismodegib after a 21‐months of follow‐up using RECIST and 60.6% for Sonidegibin 18‐month follow‐up using RECIST‐adjusted criteria.^11^ Furthermore, the panel showed a better profile for Sonidegib in terms of AEs, with 10% lower incidence than Vismodegib, possibly due to discrepancies in the pharmacokinetic profile. The importance of rotating between HHIs has also been emphasized in the Literature.^16^ Switching between HHIs may be a useful approach when considering differences in terms of AEs between the two drugs. Although AEs are not typically severe, they might be persistent, reducing patients' quality of life, and prompt treatment suspension or discontinuation. This is especially important when considering a long‐term treatment of patients with NBCCS who receive diagnosis from a young age and present a higher risk of developing locally advanced tumours. Some patients with BCC who discontinue HHI therapy due to AEs may do so because their sensitivity to the effect surpasses the amount of clinical improvement. In this sense, dermatologists should be aware of this risk and adequately manage patients to avoid treatment withdrawal. In the case of Sonidegib, dose adjustments are viable options for reducing the need for treatment discontinuation without jeopardizing its specific efficacy.^17^ Considering patients with NBCCS, a recent report showed the promising efficacy of Sonidegib in all patients treated, with partial or total clinical clearance of target BCCs, as well as pathological clearance in 57% of cases.^18^ Moreover, combination with other treatment options such as concomitant radiotherapy or even considering a neoadjuvant approach should be taken into consideration. In this case, HHI might be used to reduce primary tumour dimensions prior to excision, making it resectable. Regardless of the condition being treated, all long‐term therapy choices should be discussed with the patient within a multidisciplinary tumour board.

The aims of this report was to demonstrate the efficacy of HHI treatment with Sonidegib in patients with NCBBS who had previously treated with Vismodegib but discontinued it because of its specific AEs. As expected, the results show that Sonidegib should be considered in all aspects a first‐line treatment for patients with NBCCS not amenable for surgery or radiotherapy, and as sure a second‐line option for patients who have previously treated with other HHIs. Nevertheless, larger case studies and multicenter trials are required to validate this concept and to fully assess Sonidegib's safety and effectiveness trends seen in patients with NBCCS.

## CONFLICT OF INTEREST STATEMENT

The authors declared that they have no conflicts of interest to this work.

## AUTHOR CONTRIBUTIONS

**Federico Venturi**: Data curation (equal); Investigation (equal); Methodology (equal); Resources (equal); Writing – original draft (equal). **Luciana Trane**: Data curation (equal); Formal analysis (equal); Investigation (equal); Methodology (equal). **Flavia Silvestri**: Data curation (equal); Formal analysis (equal); Investigation (equal); Methodology (equal); Resources (equal). **Biancamaria Zuccaro**: Formal analysis (equal); Investigation (equal); Methodology (equal). **Elisabetta Magnaterra**: Data curation (equal); Formal analysis (equal); Investigation (equal); Methodology (equal); Software (equal). **Vincenzo De Giorgi**: Conceptualization (equal); Data curation (equal); Formal analysis (equal); Supervision (equal); Validation (equal); Writing – original draft (equal); Writing – review & editing (equal).

## ETHICS STATEMENT

Not applicable.

## Data Availability

The data that support the findings of this study are available from the corresponding author upon reasonable request.
